# A Numerical Method of Reduced Complexity for Simulating Vascular Hemodynamics Using Coupled 0D Lumped and 1D Wave Propagation Models

**DOI:** 10.1155/2012/156094

**Published:** 2012-05-10

**Authors:** Wilco Kroon, Wouter Huberts, Marielle Bosboom, Frans van de Vosse

**Affiliations:** ^1^Department of Surgery, Maastricht University Medical Center, 6202 AZ Maastricht, The Netherlands; ^2^Department of Biomedical Engineering, Maastricht University, P.O. Box 616, 6200 MD Maastricht, The Netherlands; ^3^Department of Biomedical Engineering, Eindhoven University of Technology, 5600 MB Eindhoven, The Netherlands

## Abstract

A computational method of reduced complexity is developed for simulating vascular hemodynamics by combination of one-dimensional (1D) wave propagation models for the blood vessels with zero-dimensional (0D) lumped models for the microcirculation. Despite the reduced dimension, current algorithms used to solve the model equations and simulate pressure and flow are rather complex, thereby limiting acceptance in the medical field. This complexity mainly arises from the methods used to combine the 1D and the 0D model equations. In this paper a numerical method is presented that no longer requires additional coupling methods and enables random combinations of 1D and 0D models using pressure as only state variable. The method is applied to a vascular tree consisting of 60 major arteries in the body and the head. Simulated results are realistic. The numerical method is stable and shows good convergence.

## 1. Introduction

Blood flow involves pressure and flow waves that propagate through the vascular system. As a compromise between computational demand and physical detail, one-dimensional (1D) network models have been used to study pressure and flow waveforms under normal and pathological conditions [1]. This modeling approach has been applied to the systemic arterial system [2–6], the coronary tree [7, 8], and the cerebral vascular tree [9, 10].

These 1D network models consist of elements that locally describe the relation between pressure and flow. Relations between pressure, area, and flow in the blood vessels are given by the 1D wave propagation equations, that is, 1D partial differential equations of mass and momentum which are derived by integrating the Navier-Stokes equation over the cross-sectional area of the blood vessel [11]. As vessel caliber decreases and the number of vessels increases towards the periphery, a point is reached where it is no longer attainable to model the vessels individually. At this point the vasculature is truncated and contribution of the distal vasculature to pressure and flow is described by 0D lumped models, such as the windkessel model [5, 6, 12] or the structured tree model [3].

To solve the system of equations derived from the 0D and 1D models and simulate propagation of pressure and flow waves through the vascular system, various, rather complex, algorithms exist. Regarding the 1D wave propagation equations, all numerical methods start from the same relation between pressure, area, and flow or cross-sectional mean velocity. First differences between the numerical methods arise with the state variables chosen to remain. With area and pressure related via a constitutive relation of the vessel wall, the result is either a pressure-flow [2, 8], area-velocity [13], area-flow [3], or pressure-velocity formulation [9, 11, 14, 15]. A second source of differences is the choice for spatial discretization of the equations. Methods include finite difference [3, 8] and spectral/finite element schemes [2, 13]. The result is a set of ordinary differential equations in which the state variables have to be solved in time. Thirdly, different methods are used to enforce continuity of pressure and flow across vessel bifurcations and at the interface between the vessels and the periphery. Methods include weak coupling of 0D and 1D equations [15, 16], computation of Riemann invariants [9, 14], or adding penalty equations [2].

The contribution of this study is to develop a simplified numerical method in which pressure is the only state variable. In this approach, the 1D wave propagation and 0D lumped model equations are cast into the same form. As such, 1D and 0D models are combined without the need to specify additional coupling equations. This allows for flexible model building from 0D and 1D elements for simulation of pressure and flow in a vascular network. For illustration, the numerical method proposed is applied to simulate pressure and flow waveforms in a vascular tree composed of 60 major arteries in the body and the head.

## 2. Method

### 2.1. Pressure-Flow Relation in Large Blood Vessels (1D)

In large arteries, blood pressure *p* (Pa), blood flow *q* (m^3^·s^−1^), wall shear stress *τ*_*w*_ (Pa), and vessel cross-sectional area *A* (m^2^) are related by 1D equations of mass and momentum. When neglecting leakage through the vessel wall as well as gravitational forces, the balance of mass and momentum is given by (derivations can be found in, e.g., Hughes and Lubliner [11] and Van de Vosse and Stergiopulos [1])

(1) $$\begin{array}{c} C_{A}\frac{\partial p}{\partial t}+\frac{\partial q}{\partial z}=0,\;\text{with}\;C_{A}=\frac{\partial A}{\partial p}, \end{array}$$

(2) $$\begin{array}{c} \frac{\rho }{A}\left(\frac{\partial q}{\partial t}+\frac{\partial }{\partial z}\left(\delta \frac{q^{2}}{A}\right)\right)+\frac{\partial p}{\partial z}=\frac{2}{a}\tau_{w}. \end{array}$$

In this (*p*, *q*, *A*) formulation, *z* (m) denotes the coordinate along the vessel axis, $a=\sqrt{A/\pi }$ (m) denotes the vessel radius, *C*_*A*_ (m^2^·Pa^−1^) denotes the vessel area-compliance, and *ρ* (kg·m^−3^) denotes the blood density.

Wall shear stress *τ*_*w*_ and constant *δ* in (2) are estimated by assuming a velocity profile. For the choice of the velocity profile, several options are possible [1]. In this study approximate velocity profiles are assumed [2]. Using approximate velocity profiles, the wall shear stress is given by

(3) $$\begin{array}{cc} & \tau_{w}=-\frac{2\eta }{\left(1-\zeta_{c}\right)a}\frac{q}{A}+\frac{a}{4}\left(1-\zeta_{c}\right)\frac{\partial p}{\partial z},\\ & \;\;\;\text{ with}\;\zeta_{c}={\left(\max\;\left[0,1-\frac{\sqrt{2}}{\alpha }\right]\right)}^{2}. \end{array}$$

with *η* (Pa·s) the blood viscosity, *ζ*_*c*_ the fraction of cross-sectional area with inertia dominated flow, and $\alpha =\sqrt{2A_{0}\rho /T\eta }$ the Womersley number that includes the duration of the cardiac cycle *T* [s], and vessel cross-sectional area *A*_0_ = *πa*_0_^2^ at reference pressure *p*_0_. For approximate velocity profiles, constant *δ* is given by

(4) $$\delta =\frac{2-2\zeta_{c}\left(1-\ln\zeta_{c}\right)}{{\left(1-\zeta_{c}\right)}^{2}}.$$

The mass and momentum equations are completed with expressions for area (*A*) and area compliance (*C*_*A*_) as a function of pressure. In this study, a nonlinear elastic vessel wall is assumed with pressure dependency of the area compliance *C*_*A*_ given by

(5) $$\begin{array}{c} C_{A}=C_{A}^{p}C_{A}^{a},\;\text{with}\;C_{A}^{p}=\beta_{1}+\beta_{2}{\left(1+{\left(\frac{p-p_{1}}{p_{2}}\right)}^{2}\right)}^{-1},\\ C_{A}^{a}=\frac{2\pi \left(1-\mu^{2}\right)a_{0}^{3}}{hE}, \end{array}$$

where *p*_1_, *p*_2_, *β*_1_, and *β*_2_ specify the pressure dependency of area-compliance (function *C*_*A*_^*p*^ from Langewouters et al. [17]). Poisson ratio *μ*, Youngs modulus *E*, and wall thickness *h* specify the radius dependency of the area compliance (function *C*_*A*_^*a*^ from Bessems et al. [2]). An expression for the pressure dependency of cross-sectional area is obtained by integrating the area compliance with respect to pressure.

### 2.2. Pressure-Flow Relation in the Periphery (0D) 

**Figure 1:**
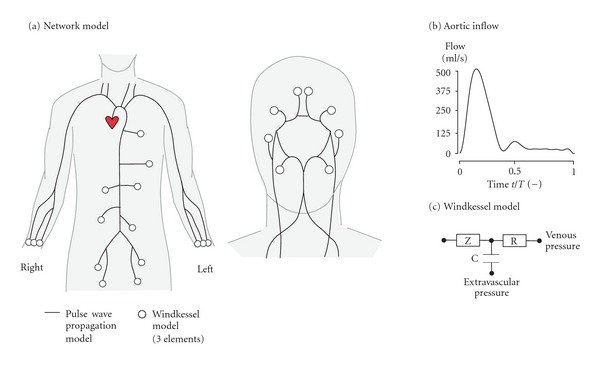
(a) Network model of 60 major arteries used to test the numerical method proposed. Adopted from Mulder et al. [10]. (b) Aortic inflow is prescribed [3]. (c) Windkessel boundary conditions at the periphery.

**Figure 2:**
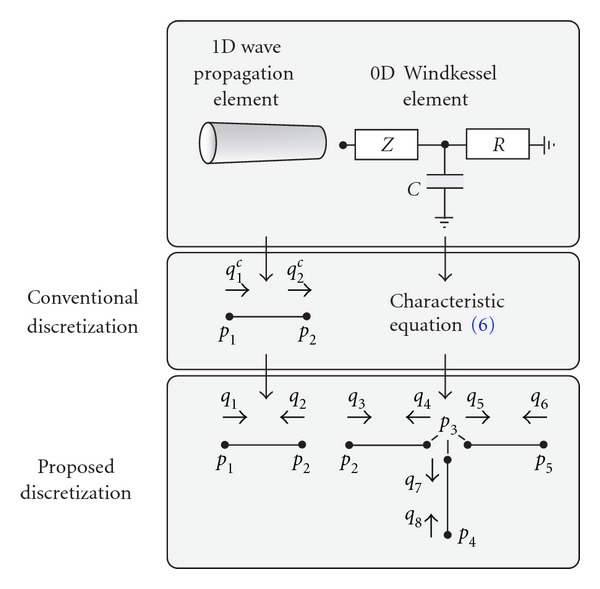
Proposed discretization for 1D wave propagation and 0D Windkessel elements. Notice the reversal of the flow in the second node with respect to the conventional discretization (indicated by superscript c).

The contribution of the peripheral vasculature at each arterial terminus is lumped in a three-element windkessel model [6, 18] (Figure 1(c)). Usually a single differential equation is derived that relates pressure *p* and flow *q* at the entrance of the Windkessel [2, 8, 10]:

(6) $$\frac{\partial q}{\partial t}=\frac{1}{Z}\frac{\partial p}{\partial t}+\frac{p}{ZRC}-\left(1+\frac{Z}{R}\right)\frac{q}{ZC},$$

with *Z* the characteristic impedance, *R* the peripheral resistance, and *C* the peripheral compliance. However, in adopting this equation, it is implicitly assumed that the venous exit pressure is zero. As such, the model's range of application is limited to that specific situation. A more general approach is to limit the Windkessel equations to those that relate pressure drops across the different elements that make up the windkessel model. Using the proposed discretization as shown in Figure 2,

(7) $$\begin{array}{c} \frac{1}{Z}\left(p_{2}-p_{3}\right)=q_{3},\;\;\frac{1}{R}\left(p_{3}-p_{5}\right)=q_{5},\\ C\left(\frac{\partial p_{3}}{\partial t}-\frac{\partial p_{4}}{\partial t}\right)=q_{7}. \end{array}\;\;$$

### 2.3. Numerical Methods

To determine pressure and flow in the vascular network, the 1D pressure-flow relations for blood vessels in (1) and (2) and the 0D pressure-flow relations for the periphery in (7) are solved in a fully coupled manner. Full coupling is achieved by casting the equations into the same discrete form.

For each of the subelements of the windkessels, two nodal point pressures and two nodal point flows are defined (Figure 2). A critical choice is that nodal point flows are both directed *inwards*. Combination of (7) with its counterpart

(8) $$\begin{array}{c} \frac{1}{Z}\left(p_{3}-p_{2}\right)=q_{4},\;\;\frac{1}{R}\left(p_{5}-p_{3}\right)=q_{6},\\ C\left(\frac{\partial p_{4}}{\partial t}-\frac{\partial p_{3}}{\partial t}\right)=q_{8} \end{array}$$

yields (Appendix A)

(9) $${\underline{C}}_{e}\frac{\partial \underset{∼}{p_{e}}}{\partial t}+{\underline{R}}_{e}^{r}\underset{∼}{p_{e}}=\underset{∼}{q_{e}},$$

where columns $\underset{∼}{p_{e}}$ and $\underset{∼}{q_{e}}$ contain the nodal point pressure and flows, respectively. Matrix ${\underline{C}}_{e}$ contains the peripheral compliance *C* and matrix ${\underline{R}}_{e}^{r}$ contains reciprocals of the characteristic impedances and peripheral resistances. A second-order backward difference scheme is used to step forward in time with time step Δ*t*. As a result the windkessel equations are written as

(10) $${\underline{K}}_{e}^{0\text{D}}\underset{∼}{p_{e}^{t+\Delta t}}=\underset{∼}{f_{e}^{0\text{D}}}+\underset{∼}{q_{e}^{t+\Delta t}},$$

with

(11) $$\begin{array}{c} {\underline{K}}_{e}^{0\text{D}}=\frac{3}{2\Delta t}{\underline{C}}_{e}+{\underline{R}}_{e}^{r},\\ \underset{∼}{f_{e}^{0\text{D}}}=-{\underline{C}}_{e}\left(-\frac{2}{\Delta t}\underset{∼}{p_{e}^{t}}+\frac{1}{2\Delta t}\underset{∼}{p_{e}^{t-\Delta t}}\right). \end{array}$$

Before, Huberts et al. [19] presented a method in which the wave propagation equations for each vessel segment were cast into a lumped model consisting of resistances, compliances, and inductances, that is, the same blocks that make up the windkessel. This approach was benchmarked with the spectral element method by Bessems et al. [2]. Here an analogous, yet more direct approach is followed without the need for deriving a lumped parameter model, leading to a simplified implementation.

First, the wave propagation equations are linearized using estimates of area compliance, cross-sectional area, wall shear stress, and convective acceleration as obtained from a previous time step (indicated by symbol $\hat{\left(•\right)}$):

(12) $$\begin{array}{c} {\hat{C}}_{A}\frac{\partial p}{\partial t}+\frac{\partial q}{\partial z}=0,\\ \frac{\rho }{\hat{A}}\frac{\partial q}{\partial t}+\frac{\partial p}{\partial z}=\frac{2}{\hat{a}}{\hat{\tau }}_{w}-\frac{\rho }{\hat{A}}\frac{\partial }{\partial z}\left(\delta \frac{{\hat{q}}^{2}}{\hat{A}}\right). \end{array}$$

Subsequently, the vessel segments are divided into smaller two-noded elements of approximate size Δ*z*. The actual size Δ*z*_*e*_ of the elements as used for the discretization can differ from Δ*z* and between vessel segments as given by

(13) $$\Delta z_{e}=\frac{l}{\max\left[\text{round}\;\left(l/\Delta z\right),1\right]}$$

with *l* the length of the vessel segment. Subsequently, a trapezium rule is used to spatially integrate the equations along the vessel axis and to express the equations in terms of nodal point pressures and flows. In this discretization step, nodal point flows for each element are also chosen to direct *inwards* (Figure 2). Once more, a second-order backward difference scheme is used to step forward in time. Consequently, mass and momentum equations are written as (Appendix B)

(14) $${\underline{K}}_{e}^{1\text{D}}\underset{∼}{p_{e}^{t+\Delta t}}=\underset{∼}{f_{e}^{1\text{D}}}+\underset{∼}{q_{e}^{t+\Delta t}}.$$

By defining both flows as being directed inwards, continuity of pressure and flow at the 0D-1D interfaces and the 1D-1D interfaces (e.g., bifurcations) is automatically satisfied in the process of assembling the element equations into the large system of equations (Appendix C). As a result, the assembled large system of equations is given by:

(15) $$\underline{K}\underset{∼}{p^{t+\Delta t}}=\underset{∼}{f}+\underset{∼}{q_{e}^{ex,t+\Delta t}},$$

where $\underset{\tilde{\;}}{q^{ex,t+\Delta t}}$ contains a zero value for each node, except for nodes where external flow is prescribed. This system of equations is solved once pressure boundary conditions and external flows are given.

Note that any nonlinearity with respect to flow makes stiffness matrix $\underline{K}$ flow dependent, thus requiring that flows are recomputed after each pressure computation. This can easily be done by using (10) or (14) at the element level; that is,

(16) $$\underset{∼}{q_{e}^{t+\Delta t}}={\underline{K}}_{e}^{\left(•\right)}\underset{∼}{p_{e}^{t+\Delta t}}-\underset{∼}{f_{e}^{\left(•\right)}},\;\left(•\right)=\left\{0\text{D},1\text{D}\right\}.$$

As such, for all elements both nodal point flows are computed.

**Figure 3:**
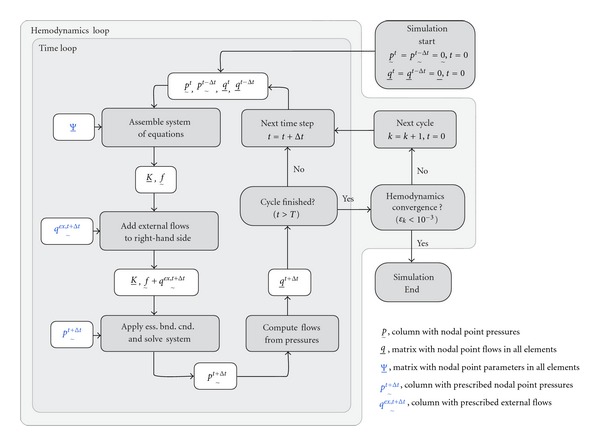
Schematic overview of the numerical method used to compute pressures and flows. During a “time loop,” a cardiac cycle with cycle time *T* is simulated with time step Δ*t*. Subsequently, a hemodynamic convergence criterion (*ε*_*k*_) is computed to assess whether pressure and flow waveforms are in steady state. If not, subsequent cardiac cycles are simulated in a “hemodynamics loop” until convergence is achieved.

After pressures and flows are computed, the simulation proceeds to the next time step. The process is repeated until cardiac cycle time *T* is reached. At this point, it is checked whether the simulation is in a hemodynamic steady state. Hemodynamic steady state is considered if the nodal point maximum relative root-mean-squared norm, denoted *ε*_*k*_, of both pressure and flow is less than 10^−3^. For *ε*_*k*_,

(17) $$\varepsilon_{k}\left(•\right)=\underset{n}{\max}\left(\sqrt{\frac{\sum_{t=0}^{t=T}{\left[{\left(•\right)}_{n,k}^{t}-{\left(•\right)}_{n,k-1}^{t}\right]}^{2}}{\sum_{t=0}^{t=T}{\left[{\left(•\right)}_{n,k-1}^{t}\right]}^{2}}}\right),\;\left(•\right)=\left\{p,q\right\},$$

with *k* the cardiac cycle number and *n* the nodal point number. A schematic overview of the complete algorithm is shown in Figure 3.

### 2.4. Simulation

#### 2.4.1. Choice of Model Parameters

The numerical method is applied to simulate hemodynamics in an arterial tree composed of the 60 major arteries in the body and the head (Figure 1(a)). Blood density *ρ* and blood viscosity *η* are assumed to be 1.05 kg·m^−3^ and 4.5 · 10^−3^ Pa·s, respectively. Reference transmural pressure (*p*_0_) is set to 13.3 kPa. Pressure dependency of the area compliance is specified according to parameters, *p*_1_ = 2.7 kPa, *p*_2_ = 4 kPa, *β*_1_ = 0.4, and *β*_2_ = 5.0 [8]. Notice that these parameters imply that at reference pressure the compliance reduces to *C*_*A*_ = *C*_*A*_^*a*^. Assuming incompressible vessel wall material renders *μ* = 0.5. Values for vessel length, wall thickness, radius, and Young's modulus as well as for the windkessel parameters are taken from Mulder et al. [10, Tables  2 and 3].

Aortic inflow is considered as the only external flow and is prescribed according to the waveform as depicted in Figure 1(b). Cardiac cycle time is set to *T* = 1 s. Venous and extravascular pressures in the windkessels are set to 0 kPa.

#### 2.4.2. Simulations Performed

To assess the convergence behavior of the proposed numerical method with respect to temporal and spatial discretization, a series of simulations is done with combinations of element sizes (Δ*z*) of approximately 2.5, 10, and 40 mm and time steps (Δ*t*) of 1, 2, 4, and 8 ms. Each simulation is started with zero pressures and flows. 

For each of the simulations, the hemodynamic convergence norm *ε* is determined as a function of cardiac cycle number. Upon convergence, pressure and flow waveforms are visualized for the aorta, the left leg, the left arm, and in the brain. The influence of spatial and temporal discretization on simulated pressure and flow waveforms is quantified by relative root-mean-square difference *ε*, as given by

(18) $$\varepsilon \left(•\right)=\underset{n}{\max}\left(\sqrt{\frac{\sum_{t=0}^{t=T}{\left[{\left(•\right)}_{n}^{t}-{\left(•\right)}_{n,\text{REF}}^{t}\right]}^{2}}{\sum_{t=0}^{t=T}{\left[{\left(•\right)}_{n,\text{REF}}^{t}\right]}^{2}}}\right),\;\left(•\right)=\left\{p,q\right\}.$$

In the computation of *ε*, waveforms as obtained with the most dense mesh and smallest time step are used as reference (indicated by subscript REF).

## 3. Results 

**Figure 4:**
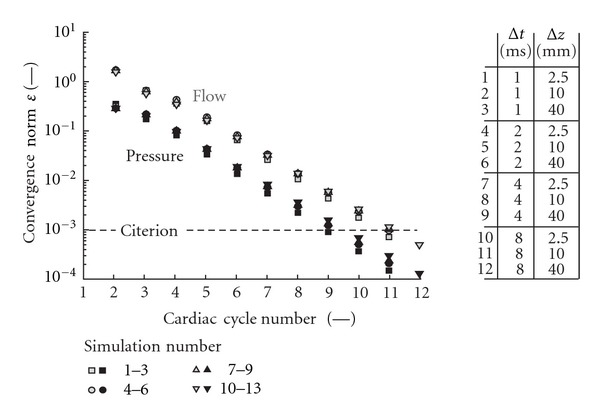
Convergence behavior for the proposed method for different time step and element sizes. Results obtained with the same time step, but different element sizes are indistinguishable.

For most simulations pressure and flow have converged after 12 cardiac cycles (Figure 4). At all intermediate cardiac cycles, the pressure norm is approximately an order of magnitude lower than that of the flow. Element size appears not to effect the decrease in *ε*-norm as a function of cardiac cycle number; that is, no visual discrimination between element sizes is possible. Convergence is slightly slower for larger time steps, but only in case of the largest time step (Δ*t* = 8.0 ms) an additional cardiac cycle is required. 

**Figure 5:**
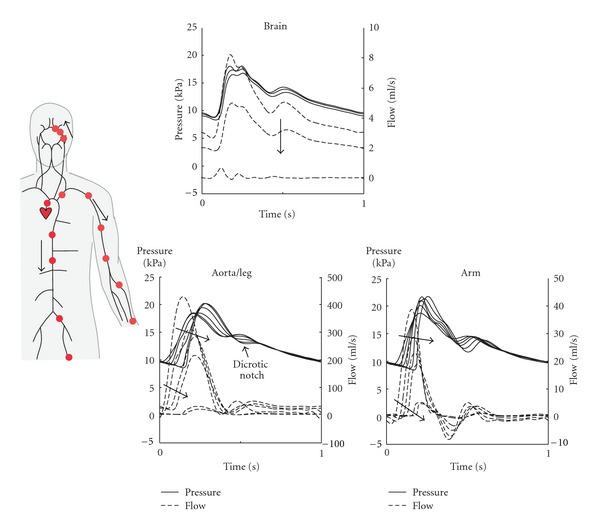
Computed pressure and flow waveforms in the aorta, the arm, and the brain. Results are shown as obtained using the simulation with element size Δ*z* = 2.5 mm and time step Δ*t* = 1 ms. The arrows indicate increase in distance from the aortic root.

Figure 5 shows the simulated pressure and flow waveforms. It is shown that the amplitude of the pressure wave increases towards the periphery. Furthermore, relative height of the dicrotic notch with respect to the pressure pulse decreases towards the periphery. Arteries near the periphery such as those in the arm show reversal of flow during a part of the cardiac cycle. 

**Figure 6:**
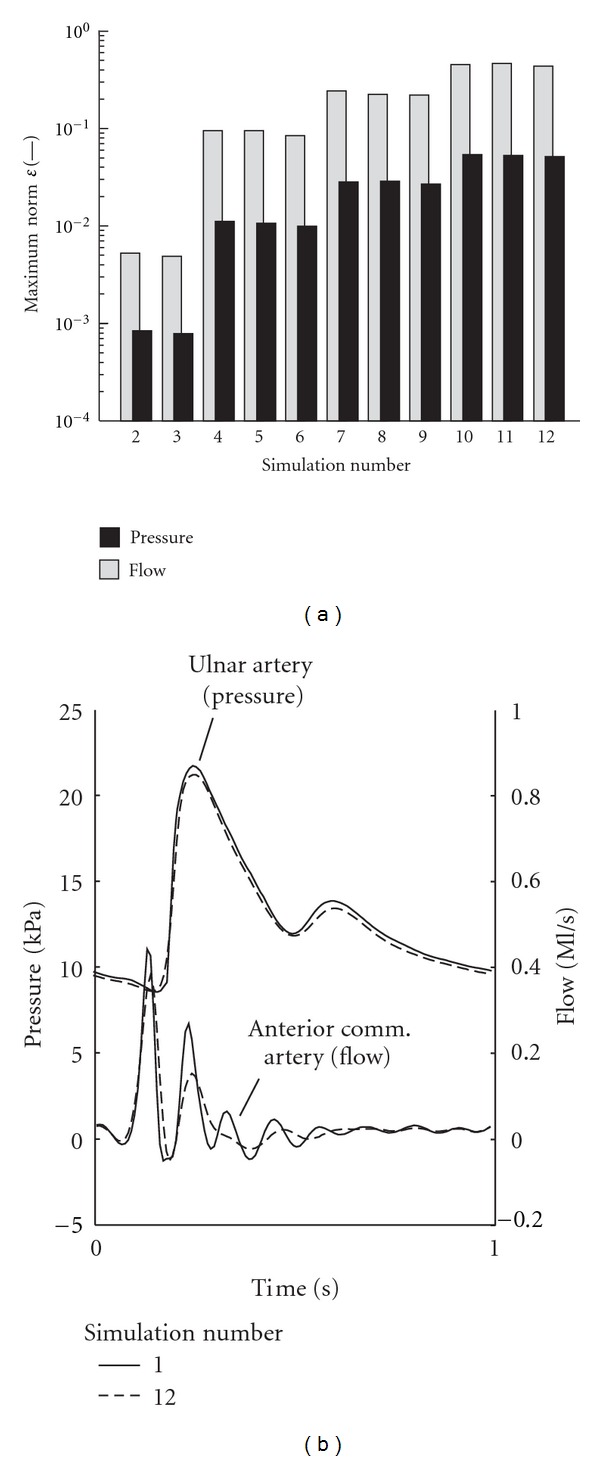
(a) Influence of element and time step size on *ε*-norm of pressure and flow. (b) Difference in pressure and flow waveform at locations with maximal *ε*-norm. The maximum *ε*-norm for pressure and flow occurs at different locations.

 As shown in Figure 6, the influence of time step size on the computed waveforms (1) is typically one order of magnitude lower for pressures than for flows and (2) increases towards the periphery; that is, the largest flow difference occurs in the anterior communicating artery in the brain and the largest pressure difference in ulnar artery in the arm (Figure 6(b)). Element size has only a minor effect on the computed pressure and flow waveforms as compared to the effect of the time step (Figure 6(a)). Using an element size of 40 mm instead of 2.5 mm at the smallest time step increases the *ε*-norm of flow by less than 5 · 10^−3^. Taking a time step of 2 ms instead of 1 ms at the smallest element size leads to an increase of approximately 8 · 10^−2^. Increasing the time step to 8 ms causes damping of the pressure and flow waveforms. 

## 4. Discussion

In this study, a simplified numerical method was developed for time-domain simulation of blood pressure and flow waveforms in the vascular system that couples nonlinear one-dimensional (1D) wave propagation models for the blood vessels to zero-dimensional (0D) lumped (windkessel) models for the periphery using pressure as degree of freedom. 

 To show performance of the method in a physiologic setting, the method was applied to simulate hemodynamics in a vascular network containing the 60 major arteries in the body and the brain. The specific choice of vessel behavior, velocity profile, windkessel parameters, and essential boundary conditions was beyond the scope of this study. The pressure-area relations of the bloodvessels were assumed non-linear and convective acceleration was included to assess behavior of the method in solving the model equations in its most non-linear form. 

The pressure and flow waveforms that were obtained with the method (Figure 5) are similar to those simulated [10] as well as experimentally measured by others [20]; that is, the computed waveforms demonstrate the physiological features of (1) increase in amplitude of the pressure wave and decrease in relative height of the dicrotic notch with increased distance from the aortic root and (2) reversal of flow during a part of the cardiac cycle in the arteries of the arm. 

### 4.1. Convergence Behavior of Proposed Method

Typically 12 cardiac cycles are needed to reach convergence when starting with zero pressure and flow conditions. This convergence is fairly independent on the element and time step size used to discretize the model equations (Figure 4). When comparing the converged situations, the time step size had a significantly larger effect on the pressure and flow waveforms than the element size (Figure 6(a)) that is, the effect of time step was typically an order of magnitude higher for flow than for pressure. Taking a time step of 8 ms introduces significant damping of the pressure and flow waves as compared to the results obtained using a time step of 1 ms (Figure 6(b)). The effect increases towards the periphery; that is, the largest root-mean-square differences are found in the ulnar artery in the arm and in the anterior communicating artery in the brain. The increase in effect of time step towards the periphery is most likely caused by the physiologic steepening of the pressure and flow waveforms towards the periphery (Figure 5). A time step of 2 ms yields an increase in relative root-mean-square difference in flow of about 8 · 10^−2^, which indicates that a time step of 1 ms is sufficiently small (Figure 6(a)).

### 4.2. Benefits of Proposed Method

As listed in the Introduction section, many different numerical methods already exist to couple the 1D wave propagation equations for the large arteries to the 0D lumped windkessel equations for the peripheral part of the vascular tree. Usually, the wave propagation equations for the vascular segments are written in discrete form using finite/spectral-element or finite-difference schemes. Such methods have the disadvantage that bifurcations require additional coupling equations to be defined in terms of the Riemann invariants or penalty functions. Furthermore, equations of the peripheral model are usually incorporated by solving a characteristic equation (such as that of the three-element windkessel in (6)) together with the wave propagation equations. The drawback of this approach is that such a characteristic equation needs to be available. This is, for instance, not the case when the terminus of the windkessels is connected to a venous circulation. 

In the numerical method proposed, the windkessel and wave propagation equations are cast into the same form to strongly couple them without the need for additional coupling equations or availability of a characteristic equation. In fact, any combination of windkessel (or lumped) elements and wave propagation elements is possible, allowing for a broader application to vascular networks that combine arteries, microcirculation (periphery), and veins. 

The numerical method allows for easy extension with a lumped model of the heart to study arterioventricular interaction such as done by others [21–23]. Cardiac contraction can be taken into account by specifying the ventricular pressure as (time-varying) essential boundary conditions, rather than prescribing the aortic inflow. Ventricular volume could then be updated during ejection using the aortic inflow of a time step earlier. Although in this way ventricular and aortic flow are only weakly coupled, numerical complexity is limited.

### 4.3. Limitations of Proposed Method

To cast equations for the wave propagation model into the same form as those for the windkessel model, it is required that each discrete element contains only two nodes in which both flows are directed inwards. As a consequence, higher-order elements such as those used in, for example, spectral element discretization are no longer possible. This limitation on order of approximation, however, was found to have little influence as element size appeared to be of minor importance for convergence as well as for the pressure and flow curves obtained. 

As indicated by the tangent of Figure 6(a), the convergence order regarding the time step is less than second order, even though a second-order backward difference scheme was used for the time integration. This reduction in convergence order can be expected due to non-linearity of the 1D wave propagation equations but may have been amplified using estimates from a previous time step for the linearization process. Linearization by means of, for example, a Newton-Raphson scheme could have been done but was not included to further simplify (implementation of) the numerical method. 

For the vascular network as presented in this study, we incorporated windkessel models for the periphery. However, the algorithm proposed is not restricted to this particular model choice. Other lumped element models such as structured tree models [3] can easily be incorporated as long as the pressure-flow relation can be cast into the same form as that for the wave propagation model. The method is also not restricted to the approximate velocity profiles as assumed in this study. Use of, for example, the Womersley velocity profiles is also possible, as the only requirement for the method to proceed in time is that area compliance, wall shear stress and convective acceleration are available at a previous time step.

## 5. Conclusion

In conclusion, a novel numerical method is developed for computation of pressure and flow waveforms in the vascular system. Using pressure as only degree of freedom, 0D lumped (windkessel) elements and 1D wave propagation elements can be randomly combined without the need for additional coupling equations. This property facilitates flexible model building from 0D and 1D elements for a wide range of applications in studying vascular hemodynamics.

## Appendices

### A. Derivation of (0D) Windkessel Element Equations

Application of definitions for nodal point pressures and flows as shown in Figure 2 yielded (7) and (8). In matrix form, these equations become

(A.1) $$\begin{array}{c} \frac{1}{Z}\underline{M}\left[\begin{array}{c} p_{2}\\ p_{3} \end{array}\right]=\left[\begin{array}{c} q_{3}\\ q_{4} \end{array}\right],\;\;\;\frac{1}{R}\underline{M}\left[\begin{array}{c} p_{3}\\ p_{5} \end{array}\right]=\left[\begin{array}{c} q_{5}\\ q_{6} \end{array}\right],\\ C\underline{M}\left[\begin{array}{c} \frac{\partial p_{3}}{\partial t}\\ \frac{\partial p_{4}}{\partial t} \end{array}\right]=\left[\begin{array}{c} q_{7}\\ q_{8} \end{array}\right], \end{array}$$

with matrix $\underline{M}$ defined as

(A.2) $$\underline{M}=\left[\begin{array}{cc} 1 & -1\\ -1 & 1 \end{array}\right].$$

Assembly of the system of equations then yields

(A.3) $${\underline{C}}_{e}\frac{\partial \underset{∼}{p_{e}}}{\partial t}+{\underline{R}}_{e}^{r}\underset{∼}{p_{e}}=\underset{∼}{q_{e}^{ex}},$$

with

(A.4) $${\underline{C}}_{e}=\left[\begin{array}{cccc} 0 & 0 & 0 & 0\\ 0 & C & -C & 0\\ 0 & 0 & 0 & 0\\ 0 & -C & C & 0 \end{array}\right],\;\;{\underline{R}}_{e}^{r}=\left[\begin{array}{cccc} \frac{1}{Z} & -\frac{1}{Z} & 0 & 0\\ -\frac{1}{Z} & \frac{1}{Z}+\frac{1}{R} & 0 & -\frac{1}{R}\\ 0 & -\frac{1}{R} & 0 & \frac{1}{R}\\ 0 & 0 & 0 & 0 \end{array}\right],$$

and $\underset{∼}{p_{e}}={\left[p_{2},p_{3},p_{4},p_{5}\right]}^{T}$, and $\underset{∼}{q_{e}}={\left[q_{3},q_{4}+q_{5}+q_{7},q_{6},q_{8}\right]}^{T}$. Since there should be no net flow to or from node 3, the choice that nodal point flows are directed *inwards* yields *q*_4_ + *q*_5_ + *q*_7_ = 0. Notice that the remaining flows *q*_3_, *q*_6_, and *q*_8_ denote *external* flows. Application of a second-order backward difference scheme yields

(A.5) $$\left[k_{0}{\underline{C}}_{e}+\underset{∼}{R_{e}^{r}}\right]\underset{∼}{p_{e}^{t+\Delta t}}=\left[{\underline{C}}_{e}\left(-k_{1}\underset{∼}{p_{e}^{t}}-k_{2}\underset{∼}{p_{e}^{t-\Delta t}}\right)\right]+\underset{∼}{q_{e}^{t+\Delta t}},$$

in which *k*_0_ = 3/(2Δ*t*), *k*_1_ = −2/Δ*t*, and  *k*_2_ = 1/(2Δ*t*).

### B. Derivation of (1D) Blood Vessel Element Equations

To describe the linearized versions of the 1D balances of mass and momentum as given by (12) in terms of a discrete number of points, the vessels are divided into a number of non-overlapping two-noded elements. As a consequence

(B.1) $$\int \left(•\right)dz\approx \overset{N_{e}}{\underset{e=1}{\sum }}\int_{e}\left(•\right)dz,$$

with *N*_*e*_ the number of elements. The trapezium rule is used to spatially integrate the element equations from node 1 to node 2. Starting with the conventional discretization with both flows directed from node 1 to node 2 as depicted in Figure 2, integration over the element domain yields

(B.2) $$\begin{array}{cc} \int_{e}{\hat{C}}_{A}\frac{\partial p}{\partial t}dz & \approx \left({\hat{C}}_{A,1}\frac{\partial p_{1}}{\partial t}+{\hat{C}}_{A,2}\frac{\partial p_{2}}{\partial t}\right)\frac{\Delta z_{e}}{2},\\ \int_{e}\frac{\partial p}{\partial z}dz & \approx p_{2}-p_{1},\\ \int_{e}{\hat{L}}_{A}\frac{\partial q}{\partial t}dz & \approx \left({\hat{L}}_{A,1}\frac{\partial q_{1}^{c}}{\partial t}+{\hat{L}}_{A,2}\frac{\partial q_{2}^{c}}{\partial t}\right)\frac{\Delta z_{e}}{2},\\ \int_{e}\frac{\partial q}{\partial z}dz & \approx q_{2}^{c}-q_{1}^{c},\;\;\int_{e}h\;dz\approx \left(h_{1}+h_{2}\right)\frac{\Delta z_{e}}{2},\\ h_{i} & ={\left(\frac{2}{\hat{a}}{\hat{\tau }}_{w}-{\hat{L}}_{A}\frac{\partial }{\partial z}\left(\delta \frac{{\hat{q}}^{c2}}{\hat{A}}\right)\right)}_{i} \end{array}$$

with Δ*z*_*e*_ the element length and *L*_*A*_ = *ρ*/*A*. Superscript c is used to indicate that conventional definition of flows are adopted. In matrix form, (B.2) becomes

(B.3) $$\begin{array}{c} {\left[\begin{array}{c} {\hat{C}}_{A,1}\frac{\Delta z_{e}}{2}\\ {\hat{C}}_{A,2}\frac{\Delta z_{e}}{2} \end{array}\right]}^{T}\frac{\partial \underset{∼}{p_{e}}}{\partial t}+{\left[\begin{array}{c} -1\\ +1 \end{array}\right]}^{T}\underset{∼}{q_{e}^{c}}=0,\\ {\left[\begin{array}{c} {\hat{L}}_{A,1}\frac{\Delta z_{e}}{2}\\ {\hat{L}}_{A,2}\frac{\Delta z_{e}}{2} \end{array}\right]}^{T}\frac{\partial \underset{∼}{q_{e}^{c}}}{\partial t}+{\left[\begin{array}{c} -1\\ +1 \end{array}\right]}^{T}\underset{∼}{p_{e}}=\left(h_{1}+h_{2}\right)\frac{\Delta z_{e}}{2}, \end{array}$$

where $\underset{∼}{p_{e}}={\left[p_{1},p_{2}\right]}^{T}$ and $\underset{∼}{q_{e}^{c}}={\left[q_{1}^{c},q_{2}^{c}\right]}^{T}$ contain nodal point pressures and flows of the element, respectively. Using the second-order backward difference scheme with time step Δ*t*, (B.3) are written as

(B.4) $${\underline{F}}_{e}\underset{∼}{p_{e}^{t+\Delta t}}+{\underline{G}}_{e}^{c}\underset{∼}{q_{e}^{c,t+\Delta t}}=\underset{∼}{h_{e}},$$

with

(B.5) $$\begin{array}{cc} {\underline{F}}_{e} & =\left[\begin{array}{cc} k_{0}{\hat{C}}_{A,1}\frac{\Delta z_{e}}{2} & k_{0}{\hat{C}}_{A,2}\frac{\Delta z_{e}}{2}\\ -1 & +1 \end{array}\right],\\ {\underline{G}}_{e}^{c} & =\left[\begin{array}{cc} -1 & +1\\ k_{0}{\hat{L}}_{A,1}\frac{\Delta z_{e}}{2} & k_{0}{\hat{L}}_{A,2}\frac{\Delta z_{e}}{2} \end{array}\right],\\ {\underset{∼}{h}}_{e} & =\left[\begin{array}{c} 0\\ \left(h_{1}+h_{2}\right)\frac{\Delta z_{e}}{2} \end{array}\right]\\ & \;+\left[\begin{array}{cc} 0 & 0\\ {\hat{L}}_{A,1}\frac{\Delta z_{e}}{2} & {\hat{L}}_{A,2}\frac{\Delta z_{e}}{2} \end{array}\right]\left(-k_{1}{\underset{∼}{q}}_{e}^{c,t}-k_{2}{\underset{∼}{q}}_{e}^{c,t-\Delta t}\right)\\ & \;+\left[\begin{array}{cc} {\hat{C}}_{A,1}\frac{\Delta z_{e}}{2} & {\hat{C}}_{A,2}\frac{\Delta z_{e}}{2}\\ 0 & 0 \end{array}\right]\left(-k_{1}{\underset{∼}{p}}_{e}^{t}-k_{2}{\underset{∼}{p}}_{e}^{t-\Delta t}\right). \end{array}$$

Next, a switch is made to the proposed discretization as illustrated in Figure 2, in which the flow in the second node is directed inwards, that is, *q*_1_ = *q*_1_^*c*^ and *q*_2_ = −*q*_2_^*c*^. Notice that this switch implies changing sign of the second column of ${\underline{G}}_{e}$. As a consequence, (B.4) becomes

(B.6) $${\underline{F}}_{e}\underset{∼}{p_{e}^{t+\Delta t}}+{\underline{G}}_{e}\underset{∼}{q_{e}^{t+\Delta t}}=\underset{∼}{h_{e}}$$

with

(B.7) $${\underline{G}}_{e}=\left[\begin{array}{cc} G_{e,11}^{c} & -G_{e,12}^{c}\\ G_{e,21}^{c} & -G_{e,22}^{c} \end{array}\right],\;\;\underset{∼}{q_{e}}=\left[\begin{array}{c} q_{1}^{c}\\ -q_{2}^{c} \end{array}\right]=\left[\begin{array}{c} q_{1}\\ q_{2} \end{array}\right].$$

After separation of matrix ${\underline{G}}_{e}$ from flow column ${\underset{∼}{q}}^{t+\Delta t}$, the (1D) element equations finally read

(B.8) $$\left[-{\underline{G}}_{e}^{-1}{\underline{F}}_{e}\right]\underset{∼}{p_{e}^{t+\Delta t}}=\left[-{\underline{G}}_{e}^{-1}\underset{∼}{h_{e}}\right]+\underset{∼}{q_{e}^{t+\Delta t}}.$$

### C. Assembly of Large System of Equations: Coupling (0D) and (1D) Element Equations

Notice that (A.5) and (B.8) are both of the form

(C.1) $${\underline{K}}_{e}\underset{∼}{p_{e}^{t+\Delta t}}=\underset{∼}{f_{e}}+\underset{∼}{q_{e}^{t+\Delta t}}.$$

Assembly of the large system of equations involves summation of all 1D element equations according to (B.1), as well as all 0D element equations. In doing so, element flows $\underset{∼}{q_{e}^{t+\Delta t}}$ will be added. As a consequence of all nodal point flows being directed inwards, for all internal nodes, the balance of mass yields a zero entry in the assembled flow column (denoted $\underset{∼}{q^{ex,t+\Delta t}}$). In fact, only non zero flows in external nodes will yield a non-zero entry in the assembled flow column. As a result, the assembled system of equations is given by

(C.2) $$\underline{K}\underset{∼}{p^{t+\Delta t}}=\underset{∼}{f}+\underset{∼}{q^{ex,t+\Delta t}}.$$

Since external flows such as that at the aortic root are prescribed, the nodal point pressures are the only degrees of freedom left, and the system can be solved once the pressures at the remaining vessel or windkessel termini are prescribed.
